# The biological modeling of autism spectrum disorders

**DOI:** 10.1192/j.eurpsy.2024.970

**Published:** 2024-08-27

**Authors:** A. Sidenkova

**Affiliations:** Psychiatry, Ural State Medical University, Yekaterinburg, Russian Federation

## Abstract

**Introduction:**

Autism Spectrum Disorders (ASD) are heterogeneous pathological conditions characterized by difficulties in establishing social contacts and the manifestation of repetitive behavior. An atypical trajectory of brain maturation, impaired neurogenesis, synaptogenesis, and an imbalance in the excitatory and inhibitory systems of the CNS form the morphofunctional basis of the ASD

**Objectives:**

scientific publications

**Methods:**

scientific analysis

**Results:**

These pathological changes appear at different stages of brain maturation They are the result of multifactorial environmental influences. To understand the functioning of this complexly organized system in time and space, a three-dimensional model is needed. The closest in vitro model of the human brain from early embryonic stages to aging is brain organoids. Human brain organoids are self-organizing three-dimensional cell aggregates derived from pluripotent stem cells. Organoids summarize neurogenesis, gliogenesis, synaptogenesis, cell migration and cell differentiation, gyrification of the cerebral cortex, reflect the connections of brain regions. The use of a 3D brain model makes it possible to simulate diseases, reactions to drugs in cells obtained from patients. The use of telencephalon organoids in the ASD model revealed that neuronal migration deficiency, acceleration and disruption of cell cycle synchronization, aberrant cell proliferation, abundant synaptogenesis, temporary deviations in the development of the cortex, increased branching of neurons, unbalanced inhibitory differentiation of neurons, high activity of ion channels are the result of impaired activity FOXG1. FOXG1 is responsible for the overproduction of GABAergic neurons. The shift towards GABAergic neurons induced by FOXG1 is positively correlated with the severity of ASD symptoms and is seen as a precursor to the future of ASD

**Conclusions:**

Thus, ASD as a socially significant disease with a heterogeneous type of inheritance, multi-link pathogenesis, realized in different periods of ontogenesis and involving different brain loci, requires special attention of researchers for the personification of diagnosis and therapy. The hiPSCs can provide insight into the cellular mechanisms underlying ASD as a neuropsychiatric disorder, providing access to the development of platforms for in vitro drug screening and patient-tailored therapy.

**Disclosure of Interest:**

None Declared

